# Annual Meeting of the Erie County Medical Society

**Published:** 1855-02

**Authors:** 


*Annual Meeting of the Erie County Medical Society.*—This meeting was largely attended, owing to the fact that it was known that Dr. J. D. Hill, a member of the society, was to be impeached for dishonorable conduct. As this action of the society possesses considerable general interest, and may become the precedent for similar action in this or other societies, we have thought it best to put on record a statement of the events and motives which led to the expulsion of Dr. Hill.

At the annual meeting of the society held in January, 1854, a committee was appointed to report to the society at its June (semi-annual) meeting, a Tariff of Prices, below which it should be dishonorable for any member to accept medical offices from the county authorities.

At the June meeting this committee reported, through their chairman, Dr. Samo. They recommended a scale of prices for each of the county medical offices. Among these the price fixed for attendance upon the county almshouse, was $600. Considering that the county buildings are five miles from the city, and that the number of patients there is usually large, (the number of deaths last year being 170) it must be conceded that the sum named by the committee was rather too low than otherwise. As an expression of the society's opinion in the premises, it was unanimously resolved that it should be dishonorable for any member to propose for or accept any of the several appointments mentioned at a sum less than that named by the Tariff of Prices.

On the 1st of October, 1854, when these appointments were made, it became known that Dr. J. D. Hill had accepted the place of Physician to the Almshouse, at $400 per annum. The society was called together at a special meeting, to consider the subject. Two other gentlemen were found to have violated the rule. Both being present expressed their regret, and pledged themselves to throw up their offices and adhere to the honor and interest of the society.

Dr. Hill was not present. Charges were preferred against him, and the society adjourned to meet some ten days thereafter.

At this second special meeting, Dr. Hill appeared and denied the right of the society to try him, affirming that the resolutions establishing the tariff of prices had not the force of a by-law, rule, or regulation. After a lengthy debate the gentleman preferring the charges withdrew them, and thus ended the controversy for the time. Before adjourning the society passed resolutions reaffirming its previous action declaring a violation of these rules dishonorable, and exhorting all honorable members to compliance with their spirit and letter. Much disappointed feeling was expressed that the society had thus failed to enforce its rules.

At the next annual meeting, held on Tuesday, 9th January, 1855, when the proper stage in the proceedings was reached, a preamble and resolution was introduced, declaring that the society would then proceed to the trial of Dr. John D. Hill, for infraction of the rules. After an animated debate this proposition was negatived by a decisive vote.

The following preamble and resolution were then introduced:

"Whereas, Dr. John D. Hill in applying for and accepting the appointment of Physician to the Almshouse for a less compensation than the rates adopted by this society, thereby avoiding fair and honorable competition, has forfeited his claims as a member of this society.

"Therefore resolved, That the said John D. Hill be, and hereby is, expelled from the Erie County Medical Society."

The discussion which followed was carried on in a tone of moderation and deference to the opinions of others, which may well be considered a proper subject for congratulation. Throughout a prolonged and animated discussion the rules of order were strictly enforced by the chair, while at its close it was evident that in the various clashings of opinion, no personal animosities had been engendered, and no remarks uttered which should have been recalled.

Without attempting to give a report of the debate, we will merely state that the arguments for expulsion were these:

No doubt existed on the part of any member, that Dr. Hill had been guilty of an infraction of the rules, in a manner pronounced dishonorable by the society.

That the society could not hope to secure a proper consideration from the county authorities, so long as it retained within its pale those members who assisted them in their resistance to the just rights of the profession.

That great injustice had been done the younger and poorer members by thus depriving them of the privilege of fair and honorable competition, and that the honor of the society required that it should punish any party thus infringing its rules, and contemning its power.

To these propositions no opposition was made, all members assenting to their correctness. The real question at issue was whether or no the society had power to expel a member thus summarily, without form of trial, and without his being notified to be present. It was strongly urged by the negative that the society should act with due foresight, that no color of persecution should be given to the transaction, and that it was unfair and illegal to expel without trial or positive proof of the offense.

The charge of illegality was met by the affirmative, by asserting that it had power to proceed to expulsion in two ways. That the form of trial under the old medical law of the state for gross immorality, or such serious charges, required a certain formality of proceedings, which was unnecessary here, inasmuch as under the old law such an expulsion stripped the person expelled of all right to practice. That, on the other hand, the society was capable to declare who should not be members, and that provision was made in its laws for a summary form of expulsion without the interference of the courts. That on a previous occasion, a person practicing homœopathy had been expelled by simple resolution, and that the courts had sustained that action. Legal precedent was, therefore, established.

The charge of unfairness was answered by the argument that the society owed a higher duty to the members who had been shut out from competition, than to the guilty party. That it had been matter of common report, that the subject was to come up at this meeting, and that Dr. Hill knowing this should appear and defend himself.

After full expression of these views, the resolution of expulsion was put under the previous question, and passed by a majority of those present.

It should be here stated that many of those voting against the resolution of expulsion, did so with the personal explanation that, were they satisfied with the legality of the mode pursued, they should vote for expulsion, and in recording their votes they wished it understood that they did not intend to justify Dr. Hill.

So Dr. John D. Hill was declared expelled from the Medical Society of the County of Erie.

It is, perhaps, not out of place for us to say here, that in our opinion the society in this action evinced a proper sense of its own rights, and a proper contempt for that system of underbidding which tends so much to make a mere trade of the honorable profession of medicine.

After the adjournment of the society its members partook of the usual annual dinner at the American Hotel. No cloud from the disagreeable duties of the day hung over the festivities of the occasion; good feeling prevailed, and a free interchange of generous sentiments betokened that the society was an united, intelligent, and efficient agency in protecting the interests confided to it.

